# Feeding di-ammonium phosphate as a phosphorous source in finishing lambs reduced excretion of phosphorus in feces without detrimental effects on animal performance

**DOI:** 10.5713/ajas.17.0591

**Published:** 2018-01-26

**Authors:** Abolfazl Koolivand, Mojtaba Yari, Saeed Khalaji, Arjan Jonker

**Affiliations:** 1 Department of Animal Sciences, Malayer University, Malayer 65719–95863, Iran; 2 Grasslands Research Centre, AgResearch Ltd. Palmerston North 4442, New Zealand

**Keywords:** Di-ammonium Phosphate, Di-calcium-phosphate, Fecal Phosphorus, Lamb Growth

## Abstract

**Objective:**

Phosphorous (P) sources with greater bioavailability might increase animal production efficiency and decrease environmental pollution. The objective of current study was to determine animal performance, nutrient digestibility, blood metabolites and fecal P concentration in finishing lambs fed a diet with either di-calcium phosphate (DCP) or di-ammonium phosphate (DAP) as a P source.

**Methods:**

Twelve 4-month-old male lambs (initial body weight 24.87±3.4 kg) were randomly allocated to a diet with either DCP or DAP (~261 g/kg of total diet P) fed *ad libitum* for 93 days. Diets were iso-nitrogenous and iso-energetic and had same calcium (Ca) and P concentrations.

**Results:**

The DAP contained 19.7 g/kg of dry matter (DM) Ca, 185.4 g/kg DM P and 14,623 ppm fluorine, while DCP contained 230.3 g/kg DM Ca, 195.2 g/kg DM P and 1,039 ppm fluorine. The diet with DAP contained 60 ppm fluorine while the diet with DCP contained 13 ppm fluorine. Lambs fed the diet with DAP tended to have a greater daily DM intake compared to those fed diet with DCP (p = 0.09). Lambs fed DAP had greater plasma P concentration and alkaline phosphatase activity (p≤0.01) compared with lambs fed DCP. Dry matter and organic matter digestibility of the diets were similar between two treatments at days 60 and 90, while they were greater in lambs fed DCP (p<0.05) at day 30 of the trial. Feeding DAP increased P digestibility (58.7% vs 50.2%; p<0.05) and decreased fecal P concentration in lambs compared with feeding DCP (3.1 vs 3.8 g/kg DM; p<0.05).

**Conclusion:**

Providing ~261 g/kg of total diet P as DAP in the diet of finishing lambs improved the bioavailability of P in the body and decreased excretion of P in feces without affecting lamb performance.

## INTRODUCTION

Phosphorus (P) is an essential mineral in the animal body (e.g. component of bone, many metabolic active compounds, genetic material and of cell structures) and therefore essential in the diet. Phosphorus is, however, an expensive mineral and represent a risk for environmental pollution [1,2]. Most of the P applied in agriculture as fertilizer or in feed is derived from phosphate rock reserves, a non-renewable resource, which are estimated to be depleted in 50 to 100 years [2]. Optimizing the supply of P in the animal diet and improving the P availability in the body when using different P sources offer an opportunity to reduce these impacts. However, deficiency in dietary P concentration or availability should be avoided to prevent metabolic disorders and poor livestock performance [3].

Di-calcium phosphate (DCP) is the common source of P in premixes for lamb rations [4]. Di-ammonium phosphate (DAP) contains very low calcium (Ca), unlike DCP, which could improve formulation of diets with high levels of legume forages that contain high Ca. Russell et al [3] and Parsad et al [5] reported that DAP can be used as a source of both P and nitrogen in ruminant diets (containing ~235.0 g/kg P and ~212.0 g/kg nitrogen). Fertilizer grade DAP might be a suitable P source for wintering diets of sheep, which are generally low in both P and N [6]. DAP is also cheaper than DCP [2]. Despite these potential beneficial properties of DAP, few studies have determined the effects of DAP in the diet of lambs. However, P sources may contain impurities (e.g. fluorine and heavy metals) that can cause toxic effects to the animal [6], which should be determined before its use in animal feeding.

We hypothesized that it would be possible to replace DCP as a common P source in the diet with DAP, without detrimental effects on the performance of growing lambs. The objectives of current study were to measure calcium, P and heavy metals concentration in DCP and DAP and to determine animal performance, nutrient digestibility, blood metabolites and fecal P concentration in finishing lambs fed a diet with either DCP or DAP as the P source.

## MATERIALS AND METHODS

### Sheep management and feeding

Twelve 4-month-old Mehraban male lambs (initial body weight [BW] 24±3.4 kg) were housed in individual cages (0.9×1.5 meter) during the current study. Lambs were vaccinated for antrotoxemia and foot and mouth disease, and orally drenched for internal parasites. A control diet was formulated according to NRC [1] recommendations and fed during the 2 weeks acclimatization period before the beginning of experiment. Feed dry matter intake (DMI) and BW were recorded during second week of this period, which were used as covariate for final statistical analysis. Lambs were randomly allocated to 2 dietary treatments containing either DCP or DAP as a P source. The both diet were formulated according to NRC [1] to have similar crude protein, metabolizable energy (ME), Ca and P concentrations (Table 1) and 261 and 305 g/kg of P in the diets was supplied by either DCP or DAP and the rest from basal diet. The diets were fed *ad libitum* (30 to 50 g/kg orts allowed) around 08:30 h every morning for 93 days. Lambs had free access to water throughout the experiment. Lambs were weighed at day 0, 21, 30, 44, 62, 78, and 93 of the experiment before morning feeding. During experiments, vitamin supplements such as A, D_3_, and E were not used in the diets due to carrier material used but they supplied through muscle injection. Animals in current study were cared for according to the guidelines of the Iranian Council of Animal Care [7].

### Sample analysis and nutrient digestibility

Fecal grab samples were collected for 4 consecutive days (one per lamb per day) at day 30, 60, and 90 at 3 h after feeding. Fecal samples were dried at 50°C for 48 h and the 4 grab samples per lamb per each sampling period were pooled. At days of fecal sampling, feed samples from forage and concentrate portion (50 g from each portion) collected and mixed in the lab to make experimental diets. Diets were oven dried at 50°C for 48 h and ground through 1 mm before further analysis. Orts were collected to measure nutrient intake. Standard procedures described by AOAC [8] were used to determine DM, ash, calcium, and P concentration. Fecal samples were analyzed for acid insoluble ash according to Van Keulen and Young [9] and organic matter (OM) according to AOAC [8]. Acid insoluble ash was used as an internal marker to estimate digestibility in combination with measured DM, OM, and P [9]. In last period of sample collection, P concentration was measured in fecal samples for 4 consecutive days. The concentrations of fluorine, cadmium, lead, mercury and arsenic of DAP, DCP, and experimental diets were measured using a ContrAA 700 (Analaytikjena, Jena, Germany) by atomic absorption method according to procedures described by Jorhem [10].

### Blood sample collection

On day 30, 62, and 93 blood samples were collected from the jugular vein of each lamb into heparinized tubes prior to feeding. Plasma Ca [11] and P [12] and serum glucose, urea nitrogen and alkaline phosphatase activity were measured using commercial kits (Pars Azmon Inc., Tehran, Iran). Glucose was measured using enzymatic photometric method [13]. Blood urea nitrogen was measured using Urease-GLDH method [14]. The alkaline phosphatase was measured using DGKC (Germany Biochemistry Association Standard) [15]. Also, photometric method by creolphthalein complex one for measuring calcium and photometric UV test for measuring P degree were used. Biochemistry experiments were performed by Auto-Analyzer machine, Hitachi 717.

### Statistical analysis

All data were analyzed by repeated measurements using Proc mixed of SAS 9.2 [16] using the following model:

$$Y_{ijk}=\mu +T_{i}+P_{j}+{lamb}_{k}+T_{i}\times P_{j}+e_{ijk}$$

Where *Y_ijk_* is the observation of dependent variable; *μ* is the fixed effect of population mean for the variable; *T_i_* is the fixed effect of supplemented P source (i = 2, DCP and DAP); *P_j_* is the fixed effect of sampling day (j = 3 for blood and fecal samples and j = 6 for DMI and BW measurements); *T_i_*×*P_j_* is the interaction between factor T at level i and factor P at level j; *lamb_k_* is the random effect of lamb within treatment (k = 6); and *e_ijk_* is the random error associated with the related observation.

The DMI and BW in the week before the beginning of the experimental treatments were included in the model as covariates. The covariate was excluded from the model when it was not significant (p>0.10). The covariance model for repeated measurements were selected based on Akaike information criterion (AIC). The adjust Tukey test was used for multiple treatment comparisons using the LSMEAN statement of SAS (SAS Institute, Cary, NC, USA). For the different statistical tests, significance was declared at p≤0.05 and trends at p≤0.10, unless otherwise stated.

## RESULTS

### Mineral and heavy metals concentration in DAP and DCP containing diets

Fertilizer grade DAP contained 19.7 and 185.4 respectively Ca and P (g/kg) and 14,623.3 ppm fluorine, while DCP contained 230.3 and 195.2 respectively Ca and P (g/kg) and 1,039.1 ppm fluorine (Table 1). The DCP contained more lead, while concentration of mercury, cadmium and arsenic were similar compared with DAP. The solubility of P in acid was similar for DAP and DCP. The diet with DAP contained 60 ppm fluorine while the diet with DCP contained 13 ppm fluorine (Table 2). Both experimental diets had similar Ca, P, and other nutrients measured (Table 2).

### Animal performance

Lambs fed the diet with DAP tended to have greater daily DM intake during experiment compared to those fed the diet with DCP (p = 0.09; Table 3). Average daily gain, final BW, BW change and feed conversion ratio were similar between lambs fed the two dietary treatments. Daily DM intake and BW increased with advancing lamb age (p<0.05). Average daily gain and feed conversion ratio remained similar over the 93 days measurement period.

There was a dietary treatment×time period interaction (p < 0.05; Table 4) for DM and organic matter digestibility. At day 30, DM and organic matter digestibility were greater in lambs fed the diet with DCP compared with DAP (p<0.05), while at days 60 and 90 of the trial, DM and organic matter digestibility were similar for lambs fed the two dietary treatments. Fecal P concentration was lower for lambs fed the diet with DAP than for lambs fed the diet with DCP at day 90 of the experiment (p<0.05; Table 4). Apparent digestibility of P was greater for lambs fed the DAP diet compared with lambs fed DCP diet (p<0.05; Table 4).

### Blood metabolites

Serum urea and plasma Ca were similar between lambs fed diets with DAP and DCP (Table 5). Feeding the diet with DAP increased plasma P and serum alkaline phosphatase enzyme (p<0.05) compared with feeding the diet with DCP. There was a dietary treatment×time period interaction (p<0.05) for serum glucose. At day 60 of feeding DAP, serum glucose was greater compared with feeding DCP (p<0.05), while serum glucose was similar at the other sampling times for lambs fed either DAP or DCP (Table 5). Blood metabolites changed as the age of lambs increased (Table 5). Lambs had lower blood glucose at day 90 compared with days 30 and 60 (p<0.0001), lower blood urea and P at day 60 compared with days 30 and 90 (p<0.0001) and higher blood alkaline phosphatase concentration at day 90 compared with day 30 and 60 (p<0.0001). Lambs had lower blood Ca at day 30 compared with day 90, which were both lower than on day 60 (p<0.0001).

## DISCUSSION

Lambs fed the diet with DAP tended to have a greater DM intake than those fed the diet with DCP in the current study, which was opposite to findings of Oltjen et al [6] who reported that feeding DAP decreased DM intake of finishing lambs. The DAP source used in the current study was in a powder form while DAP was in a granule form in the study of Oltjen et al [6]. Adding DAP as powder likely improved mixing with the concentrates, which might improve the diet palatability and reduce feed selection and sorting by sheep. Oltjen et al [6] reported that including DAP in pelleted concentrate rather than in granule form would increase its intake and palatability. Further, only 261 g/kg of total P of diet was supplied by DAP in current study, while Oltjen et al [6] supplied 940 g/kg of P in the diet as DAP. The amount of DAP supplemented in the diet might affect the palatability of the diet. Phosphorus is involved also in the control of appetite (in a manner not yet fully understood) and in the efficiency of feed utilization [17].

Average daily gain and feed conversion ratio were similar for lambs fed DAP and DCP in the current study. Venediktove et al [18] reported that feeding a diet supplemented with DAP improved weight gain and feed efficiency in calves compared with feeding DCP and Karadzyan et al [19] reported that feeding DAP improved weight gain in steers. Karadzyan et al [19] indicated that P from DAP had greater bioavailability than P from DCP, which was also found in the current study with lambs fed with DAP having lower fecal P and higher plasma P, which indicated that P from DAP had a greater bioavailability. Phosphorus concentration in feces is affected by endogenous losses and amount of P intake [2,20,21] and plasma P is strongly related to amount of P intake and the bioavailability of P resources [4,15]. The DM intake of lambs fed DAP and DCP were similar and therefore the difference in fecal and plasma P concentrations were likely not affected by level of intake. Annenkove et al [22], Bogdani et al [23] and Suttle [2] reported also higher bioavailability of P from DAP compared with P from DCP.

The diet ingredients provided 739 and 695 g/kg of P lamb requirements and 261 and 305 g/kg of P requirements were provided by DAP and DCP respectively. The lack of lamb performance responses in the current study might be due to the low inclusion level of DAP and DCP or lamb requirements might have been met independent of bioavailability. A phosphorus deficiency may be observed by slow growth, declined appetite, unthrifty appearance, listlessness, low level of phosphorus in blood (less than 4 mg/dL of plasma), and development of rickets [4], which were all not the case with either P supplement in the diet. Further, digestibility of DM and organic matter was lower in lambs fed DAP than in lambs fed DCP (648.0 vs 690.0 and 681.0 vs 723.0 g/kg) at the end of first period (30 d), which was likely due to the greater DM intake of lambs fed DAP that can result in decreased nutrient digestibility [4]. Energy intake and availability were therefore likely similar between the two diets, another likely reason why lamb performance was similar.

Feeding DAP increased serum alkaline phosphatase in lambs. Alkaline phosphatase activity gives a good indication of the extent of new bone formation and osteoblast activity [24] and was found to be decreased under poor feeding conditions in young sheep, which may influence growth rate [25].

One concern about using of fertilizer grade DAP is its flu orine content, which if fed at high levels can exerts a cumulative toxic effect. Oltjen et al [6] reported that feeding 88 grams DAP (fluorine concentration not provided) in the diet of sheep with a BW of 45 kg resulted in toxic effects. Phosphorus to fluorine ratio in DAP used in the current study was 12.7, which was lower than the threshold of 120 recommended for minimizing the risk of fluorosis toxicity when using P sources [4,2]. With the inclusion of DAP to provide 261 g/kg of total P in the diet, however, the fluorine concentration of diet reached 60 ppm which has been reported as the maximum tolerable level for breeding sheep [4]. A de-fluorination procedure may decrease the fluorine concentration in DAP. Phosphorus resources can also contain heavy metals at toxic levels, which can be hazardous for the animal health [2]. The concentration of potential toxic elements in diets with either DAP or DCP in the current study were, however, below hazardous threshold levels for ruminants [26] and will therefore be safe to feed at the DAP and DCP inclusion levels used.

## CONCLUSION

DAP had lower P and higher N and fluorine compared with DCP. Using DAP to supply 261 g/kg of total diet phosphorous instead of DCP did not affect the growth and feed conversion efficiency in finishing lams. Greater plasma P and lower fecal P concentration in lambs fed DAP indicate greater P bioavailability from DAP than from DCP in finishing lambs.

## Figures and Tables

**Table 1:** Macro mineral (g/kg) and heavy metals (ppm) profile of di-calcium phosphate, di-ammonium phosphate and calcium carbonate used in the experimental diets of finishing lambs

Items	Ca	P	F	Pb	Hg	Cd	As	P:F	ASP
DCP	230.3	195.2	1,039.1	41.7	1.8	2.8	7.6	1.9	97.5
DAP	19.7	185.4	14,623.3	3.8	1.7	2.7	6.5	12.7	96.6
CaCO_3_	390.2	6.0	22.4	17.4	15.6	2.9	9.5	27.3	ND

P:F, ratio of phosphorous to fluorine; ASP, acid soluble phosphorus; DCP, di-calcium phosphate; DAP, di-ammonium phosphate; ND, not determined.

**Table 2:** Ingredients and chemical composition of experimental diets fed to finishing lambs

Items	DCP	DAP
Ingredients (g/kg of dry matter)		
Wheat straw	350	350
Beet pulp	320	320
Corn grain	170	170
Barley grain	100	100
Soy bean meal	50	50
DAP	-	3.45
DCP	3.83	-
Urea	2.95	1.56
CaCO_3_	-	2.15
NaCl	2.50	2.50
Filler	0.72	0.34
Nutrients profile and chemical composition (g/kg of dry matter)1)		
Metabolisable energy (Mcal/kg DM)1)	2.38	2.38
Crude protein1)	106	106
Acid detergent fiber1)	325	325
Neutral detergent fiber1)	455	455
Ether extract1)	18.4	18.4
Non-fiber carbohydrates1)	356	356
Ash	65.6	65.3
Calcium	3.59	3.59
Phosphorus	2.45	2.45
Calcium:phosphorus	1.47	1.47
P from DAP or DCP (g/kg of total P)	261	305
Heavy metals (ppm)		
F	13.0	60.0
Pb	0.57	0.46
Hg	0.06	0.09
Cd	0.09	0.09
As	0.88	0.89

DCP, di-calcium phosphate; DAP, di-ammonium phosphate.

1) Calculated according to feed chemical composition data in NRC [1] tables.

**Table 3:** Dry matter intake (DMI) and performance of finishing lambs fed a diet supplemented with either di-calcium phosphate (DCP) or di-ammonium phosphate (DAP) as the phosphorous source during a 93 day period

Items	Parameters				

	DMI (kg/d)	ADG (kg/d)	BW (kg)	BWC (kg)	FCR (kg DM/kg ADG)
Treatments					
DCP	1.16	0.161	33.85	2.48	8.44
DAP	1.22	0.182	34.37	2.79	7.25
SEM	0.023	0.0108	0.248	0.152	0.603
p-value	0.09	0.17	0.15	0.18	0.17
Periods (d)					
0–15	0.98^c^	0.142	28.00^f^	2.98^ab^	7.30
16–30	1.08^c^	0.167	29.50^e^	1.51^c^	6.79
31–45	1.15^b^	0.173	31.91^d^	2.42^b^	8.55
46–60	1.16^b^	0.194	35.73^c^	3.50^a^	6.53
61–75	1.33^a^	0.161	38.33^b^	2.57^b^	9.35
76–90	1.43^b^	0.189	41.19^a^	2.84^ab^	8.36
SEM	0.039	0.0178	0.413	0.252	1.045
p-value	<0.0001	0.36	<0.0001	<0.0001	0.32
Treatment×period					
p-value	0.87	0.93	0.99	0.91	0.64

ADG, average daily gain; BWC, body weight change; FCR, feed conversion ratio calculated as dry matter intake/weight gain; SEM, standard error of means.

Means with different letter (a–f) within column differ p<0.05.

**Table 4:** Dry mater, organic matter and phosphorous apparent total tract digestibility (%) and fecal phosphorous concentration (g/kg DM) of finishing lambs fed a diet supplemented with either di-calcium phosphate (DCP) or di-ammonium phosphate (DAP) as the phosphorous source

Treatments	Parameters			

	Dry matter digestibility	Organic matter digestibility	Fecal phosphorous concentration	Phosphorous digestibility
DCP	67.9	71.2	3.8^a^	50.2^a^
DAP	66.8	70.2	3.1^b^	58.7^b^
SEM	0.59	0.62	0.18	1.85
p-value	0.19	0.25	<0.05	<0.05
Days of sampling				
30	669	702	-	-
60	682	713	-	-
90	669	707	-	-
SEM	7.2	7.6	-	-
p-value	0.35	0.76	-	-
Treat×time1)	<0.01	<0.05	-	-

SEM, standard error of means.

1) At day 30, lambs fed with DCP had greater dry matter and organic matter digestibility (p<0.05).

Means with different letter (a,b) within column differ p<0.05.

**Table 5:** Blood metabolite profile in finishing lambs fed a diet with either di-calcium phosphate (DCP) or di-ammonium phosphate (DAP) as a P source during 93 days

Items	Glucose (mg/dL)	Urea (mg/dL)	Calcium (mg/dL)	Phosphorous (mg/dL)	Alkaline phosphatase (IU/L)
Treatments					
DCP	59.42	8.74	9.03	4.76^b^	626.7^b^
DAP	61.06	8.28	8.96	4.99^a^	741.2^a^
SEM	1.536	0.53	0.068	0.071	35.36
p-value	0.46	0.54	0.42	0.03	0.03
Day of sampling					
30 d	65.67^a^	10.92^a^	8.20^c^	5.44^a^	647.7^b^
62 d	69.70^a^	6.57^b^	9.54^a^	3.71^b^	606.1^b^
93 d	45.35^b^	7.87^a^	9.24^b^	5.47^a^	898.1^a^
SEM	1.882	0.649	0.083	0.088	41.31
p-value	<0.0001	<0.0001	<0.0001	<0.0001	0.01
Treat×time	0.043	0.33	0.75	0.09	0.57

SEM, standard error of means.

Means with different letter (a–c) within column differ p<0.05.
